# Saphenous Vein Graft Failure: Current Challenges and a Review of the Contemporary Percutaneous Options for Management

**DOI:** 10.3390/jcm12227118

**Published:** 2023-11-15

**Authors:** Liam Back, Andrew Ladwiniec

**Affiliations:** Glenfield Hospital, Leicester LE39QP, UK; andrew.ladwiniec@uhl-tr.nhs.uk

**Keywords:** saphenous vein graft, coronary artery bypass graft, percutaneous coronary intervention

## Abstract

The use of saphenous vein grafts (SVGs) in the surgical management of obstructive coronary artery disease remains high despite a growing understanding of their limitations in longevity. In contemporary practice, approximately 95% of patients receive one SVG in addition to a left internal mammary artery (LIMA) graft. The precise patency rates for SVGs vary widely in the literature, with estimates of up to 61% failure rate at greater than 10 years of follow-up. SVGs are known to progressively degenerate over time and, even if they remain patent, demonstrate marked accelerated atherosclerosis. Multiple studies have demonstrated a marked acceleration of atherosclerosis in bypassed native coronary arteries compared to non-bypassed arteries, which predisposes to a high number of native chronic total occlusions (CTOs) and subsequent procedural challenges when managing graft failure. Patients with failing SVGs frequently require revascularisation to previously grafted territories, with estimates of 13% of CABG patients requiring an additional revascularisation procedure within 10 years. Redo CABG confers a significantly higher risk of in-hospital mortality and, as such, percutaneous coronary intervention (PCI) has become the favoured strategy for revascularisation in SVG failure. Percutaneous treatment of a degenerative SVG provides unique challenges secondary to a tendency for frequent superimposed thrombi on critical graft stenoses, friable lesions with marked potential for distal embolization and subsequent no-reflow phenomena, and high rates of peri-procedural myocardial infarction (MI). Furthermore, the rates of restenosis within SVG stents are disproportionately higher than native vessel PCI despite the advances in drug-eluting stent (DES) technology. The alternative to SVG PCI in failed grafts is PCI to the native vessel, ‘replacing’ the grafts and restoring patency within the previously grafted coronary artery, with or without occluding the donor graft. This strategy has additional challenges to de novo coronary artery PCI, however, due to the high burden of complex atherosclerotic lesion morphology, extensive coronary calcification, and the high incidence of CTO. Large patient cohort studies have reported worse short- and long-term outcomes with SVG PCI compared to native vessel PCI. The PROCTOR trial is a large and randomised control trial aimed at assessing the superiority of native vessel PCI versus vein graft PCI in patients with prior CABG awaiting results. This review article will explore the complexities of SVG failure and assess the contemporary evidence in guiding optimum percutaneous interventional strategy.

## 1. Introduction

Coronary artery bypass grafting (CABG) remains an important and highly prevalent management option in the contemporary treatment of obstructive coronary artery disease. Despite a noted decline in CABG frequency in the UK since 2014 [1], current European guidelines advocate CABG in preference to percutaneous coronary intervention (PCI) in multiple coronary syndromes including left main disease (Level 1, Class A) and three vessel coronary disease with an intermediate to high SYNTAX score, regardless of the presence of diabetes mellitus (Level 1, Class A) [2]. Arterial conduits in contrast to venous have long been demonstrated to provide a superior and durable graft result with a left internal mammary artery graft (LIMA) maintaining a patency rate of 88–100% at 15 years [3,4,5,6] and meta-analyses demonstrating a significantly higher saphenous venous graft (SVG) failure rate when compared to radial arterial grafts [7]. Despite this growing understanding of their limitations in longevity, the use of SVGs as a surgical conduit in CABG remains high [8,9,10,11,12,13,14,15,16,17]. As SVGs do not mirror the natural history of arterial grafts, with an observed accelerated rate of atherosclerosis in both the conduit and the native vessel, they present unique challenges with regards to the management of graft failure in a population with a substantial lifespan after their initial revascularisation. This review article will explore the contemporary challenges of SVG failure and discuss the percutaneous options for management in this patient cohort.

## 2. Choice of Surgical Conduit

### Arterial versus Venous Conduit in Coronary Artery Bypass Grafting

The use of a LIMA graft to the left anterior descending artery (LAD) was established in 1986 following a landmark study demonstrating improved mortality in coronary revascularisation and remains the cornerstone of surgical coronary artery disease management worldwide [18]. Which additional conduit is the optimum vessel for surgical management has been an important consideration for cardiac surgeons, with both arterial and venous conduits having been studied extensively. Several trials have demonstrated higher rates of angiographic patency in radial artery (RA) grafts versus SVG; however, these trials were underpowered to detect differences in the frequency of clinical events [19]. While the superiority of RA over SVG has been supported in a recent meta-analysis [20], with lower rates of adverse cardiac events and occlusion at five years, there remains a reluctance in the wider surgical community to adopt the practice of multiple arterial grafts. As such, due to its accessibility and length, the greater saphenous vein remains the most widely utilised conduit and the consequence of its limited longevity remains an important clinical consideration.

## 3. Saphenous Vein Graft Failure

### 3.1. The Natural History of Saphenous Venous Grafts Following Anastomosis

In contemporary CABG, up to 95% of patients worldwide receive at least one SVG in addition to the use of a LIMA [12,21]. The precise patency rates for SVG vary widely in the literature, with estimates of 11–41% failure rate at less than 3 years [8,9,13,14], 19–33% failure rate at 5 to 10 years [10,15,16,17], and 39–61% at greater than 10-year follow-up [10,11,22,23,24] (Table 1). SVGs are known to progressively degenerate over time and, even if they remain patent, demonstrate marked accelerated atherosclerosis. As early as the first year following anastomosis to the arterial system, neointimal hyperplasia and foamy macrophages are observed in SVGs, progressing to stenotic lesions through the expansion of necrotic cores [25]. Within 5 to 10 years, these neoatherosclerotic plaques are well established and often associated with friability, erosive features, and luminal thrombus [25,26]. This leads to the high occurrence of ischaemia-driven events, distal embolisation, and occlusive pathology.

### 3.2. The Natural History of Native Artery Coronary Atherosclerosis Following Grafting

**Figure 1:**
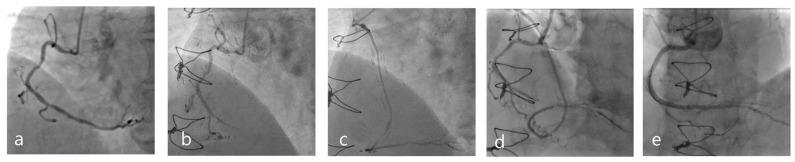
A 72-year-old diabetic gentleman presented acutely to our facility with a non-ST segment myocardial infarction (NSTEMI). Index coronary angiography revealed three-vessel disease, with severe diffuse left anterior descending (LAD) disease, severe focal first obtuse marginal (OM1) disease, and moderate ostial, mid, and distal right coronary artery (RCA) disease, with an invasive fractional flow reserve (FFR) of 0.80 (**a**). He underwent coronary artery bypass grafting (CABG) with a LIMA to his mid-LAD and a saphenous vein graft (SVG) to his distal RCA, with no suitable graft target identified for his OM1. Eight months following his CABG, this gentleman represented acutely to our facility with another NSTEMI. Repeat coronary angiography demonstrated rapid progression of his RCA disease, with a now occluded distal RCA (**b**). In the same period, his SVG to RCA had degenerated significantly, with a long segment stenosis in its mid-segment, with further sequential severe lesions in its distal third (**c**). This gentleman was referred for chronic total occlusion (CTO) PCI, with contralateral injections performed during this procedure demonstrating the extent of progression of native RCA disease with an occluded distal RCA (**d**). His native RCA was recanalized percutaneously with drug-eluting stents to replace his failing SVG with a good angiographic result (**e**).

Before considering the options available for the management of SVG failure, it is important to consider the natural history of the native coronaries following graft implantation. Although the majority of evidence investigating this issue preceded the widespread use of internal mammary grafts and statins, multiple studies have demonstrated a marked acceleration of atherosclerosis in bypassed arteries compared to non-bypassed arteries [27,28,29,30]; this phenomenon is frequently encountered in clinical practice (Figure 1). In one study, the progression of atherosclerosis, defined as at least a further 25% lumen loss during a mean follow-up period of 37 months, was more than 10 times as frequent in bypassed arteries with minimal atherosclerosis as compared to non-bypasses arteries [28]. The status of the bypassed native coronary artery, particularly progression to a chronic total occlusion (CTO), is clinically important when considering graft failure as multicentre registry data demonstrate a lower technical rate of success in revascularisation of CTO patients with prior CABG compared to those without [31,32,33].

## 4. Diagnosis of Graft Failure

As the expected prognosis of patients post-CABG has improved significantly over the past three decades [34,35], the diagnosis and management of SVG failure will become an increasingly important issue in cardiac surgical and cardiology practice. The need for repeat revascularisation procedures following CABG is known to increase cumulatively over time, with registry data suggesting a revascularisation rate of 2% in the first year following primary CABG, 7% at 5 years, 13% at 10 years, and 16% at 18 years [36] (Table 2). While the investigation of graft failure remains similar to the investigation of de novo ischaemic heart disease syndromes, with invasive coronary angiography (ICA) serving as the definitive investigation, graft evaluation on these patients confers additional challenges. These challenges can include a higher degree of calcification, higher number of vessels to engage, variability in the ostia positioning of grafts, and uncertain or incomplete operative information prior to the procedure. For these reasons, invasive coronary angiography in patients with previous CABG is known to lead to longer procedure times, higher total contrast dose and radiation exposure, and increased risk of major complications including stroke and contrast-induced nephropathies when compared to patients without prior CABG [37,38,39,40].

Research into alternative investigative strategies for saphenous vein graft failure, particularly CT coronary angiography (CTCA), has been promising in minimising these additional risks. CTCA has been demonstrated to be highly effective in detecting graft stenoses, with observational data estimating both sensitivity and specificity greater than 95% [41,42]. The BYPASS-CTCA trial, randomising 688 patients with prior CABG to CTCA prior to ICA versus ICA alone, was able to demonstrate reductions in procedural times (18.6 ± 9.5 min versus 39.5 ± 16.9 min (*p* < 0.001)), contrast nephropathy (3.4% versus 27.9%, *p* < 0.001), and improved patient satisfaction with this strategy [43]. Additionally, 1-year major adverse cardiac events were lower in the CTCA group compared to the ICA alone group (16.0% versus 29.4%, *p* < 0.001). These data suggest that the use of complementary imaging modalities to ICA including CTCA will be highly important in the management of saphenous vein graft failure and a potential future direction for improving clinical outcomes.

## 5. Management of Graft Failure

### 5.1. Medical Therapies to Improve Saphenous Vein Graft Patency

Several medical therapies are well established with regards to improving SVG patency post CABG. The use of aspirin early post CABG is critical to improving graft patency, with up to five times increased frequency of failure in patients not treated postoperatively [44]. The addition of P2Y12 inhibitors, vitamin K antagonists, or novel anticoagulant therapy with aspirin has not demonstrated any consistent benefit to SVG patency and is not routinely adopted in practice [45,46,47,48,49,50]. Statin therapy has been demonstrated to reduce SVG occlusion rates in addition to adverse cardiac events following CABG [51,52]. The addition of ezetimibe in patients with prior CABG may attenuate the benefits of statin therapy with regards to SVG failure [53]. There is growing interest in the intensity of lipid lowering therapy and its effect on SVG failure and the potential benefit of PCSK9 inhibition. An aggressive LDL target of <2.6 mmol/L to prevent SVG disease was established in the post-coronary artery bypass graft trial and supported by a post hoc analysis of the clopidogrel after surgery for coronary artery disease (CASCADE) trial [51,54]. Furthermore, in a recent study of 231 prior CABG patients, higher circulating PCSK9 levels were associated with a higher risk for SVG occlusion after adjustment for conventional cardiovascular risk factors [55]. Several studies await the investigation of the effect of PCSK9 inhibition on SVG patency, disease rate, and occlusion and will provide important insight into the optimisation of medical therapy for these patients (NCT03900026, NCT03542110). While medical therapy is often indicated in the treatment of graft failure, many patients will present with unstable coronary syndromes or refractory symptoms and hence mandate an appropriate revascularisation procedure.

### 5.2. Redo CABG in the Context of Failed Saphenous Vein Grafts

The incidence of redo CABG procedures has been decreasing consistently over the past two decades in preference for percutaneous interventional techniques. Of the CABG procedures reported to the Society of Thoracic Surgeons (STS) adult cardiac surgery database, as a percentage of overall CABG volume, redo CABG had decreased from 6.4% in 2000 to 3.6% in 2009 and to 2% in 2017 [56,57]. Redo CABG has a multitude of additional challenges including cardiovascular injury on sternal reentry, a lack of graft conduits, and limitation of arterial cannulation and cross-clamping for cardiopulmonary bypass secondary to mediastinal adhesions [58]. Compared with primary CABG, hospitalisations for redo CABG were associated with a statistically significant higher in-hospital mortality of 3.2% versus 1.9% [59]. For these reasons, PCI following graft failure has gained favour over redo CABG as the initial treatment strategy [60].

### 5.3. Percutaneous Coronary Intervention in the Context of Failed Saphenous Vein Grafts

The percutaneous options for the management of a failing SVG include PCI to the venous conduit and PCI to the native vessel. Although traditionally PCI to the venous conduit has been the primary treatment strategy, a growing understanding and familiarity with complex techniques including CTO PCI has expanded options for PCI to the native artery, making this a viable strategy for revascularisation when required. Studies are ongoing with regards to the superiority of this strategy in comparison to SVG PCI (NCT03805048).

### 5.4. Percutaneous Coronary Intervention to a Failing Saphenous Vein Graft

Percutaneous treatment of a degenerative SVG provides unique challenges secondary to a tendency for frequent superimposed thrombus on critical graft stenoses, friable lesions with marked potential for distal embolisation and subsequent no-reflow phenomena, and high rates of peri-procedural myocardial infarction (MI). Estimates of no-reflow phenomena range between 6 and 10% in SVG PCI compared with 1 and 2% in native vessel PCI despite variable use of intracoronary embolic protection devices (EPD) in these studies [61,62]. The use of EPD in SVG PCI is an important consideration when attempting to mitigate the risk of distal embolisation. While their use is endorsed as a Class I recommendation by both European and American contemporary guidelines for SVG intervention [63,64], this is guided primarily by a single randomised controlled trial conducted on the first commercially available device. The Percusurge Guardwire System (Medtronic, Santa Rosa, California) was the first widely available EPD, consisting of a compliant balloon advanced distal to the SVG lesion to occlude flow and an aspiration catheter to remove embolic debris [65]. This device was evaluated in the SAFER trial (saphenous vein graft angioplasty free of emboli randomised) where it was recognised to lead to a 42% reduction in the primary composite endpoint of death, MI, emergency CABG, or target vessel revascularisation at 30 days [65]. This finding was noted to be primarily driven by a 46% reduction in periprocedural MI. Since this time, several studies, including data from a large-scale National Cardiovascular Data Registry (NCDR-CathPCI Registry) have found conflicting results, with no additional benefit demonstrated with routine use of EPD during SVG intervention [65,66,67,68]. An additional significant association between the use of EPD and periprocedural complications has been demonstrated in three-year registry data [69]. Conversely, a recent meta-analysis of 11 studies including over 79,000 patients demonstrated a significantly lower odds ratio of mortality with the routine use of EPD compared with standard therapy in SVG PCI (OR 0.69, 95% CI 0.5–0.94), with no significant difference with regards to MACE, target vessel revascularisation, or periprocedural and late MI [70]. The use of EPD in SVG PCI continues to be a potential consideration to reduce the established higher risk of this intervention.

**Figure 2:**
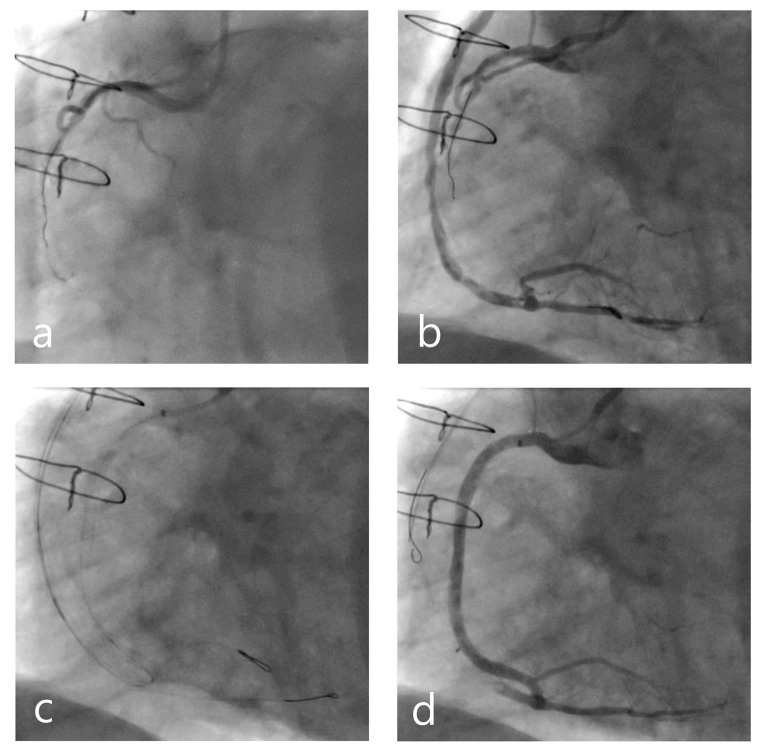
An 80-year-old diabetic gentleman with a background of remote coronary artery bypass grafting (CABG) in 1997 (LIMA-LAD, SVG-OM, and SVG-RCA) and PCI to SVG-RCA in 2020 presented acutely to our facility with an inferior ST-segment elevation myocardial infarction (STEMI). He was transferred for emergency coronary angiography where his culprit lesion was identified as his SVG to RCA which had occluded proximal to his previous drug-eluting stent (DES). His native RCA was chronically occluded in the mid-segment (**a**). He underwent thrombus aspiration and balloon angioplasty to the SVG with adequate antegrade flow restored. In discussion with our local complex total occlusion (CTO) operators, this gentleman was referred for urgent staged CTO PCI to his native RCA in preference of repeat SVG PCI. Contralateral injections during the CTO procedure demonstrated a long segment mid-RCA CTO in addition to a diffusely diseased SVG, with a severe lesion in the mid-segment and residual heavy thrombus burden despite GPIIb/IIIa inhibition and thrombus aspiration (**b**). Using the degenerated venous conduit, retrograde access was achieved to the CTO segment where reverse controlled antegrade and retrograde tracking (reverse-CART) and wire externalisation were able to be achieved (**c**). The native RCA was subsequently recanalized with DES to replace the failing SVG with a good angiographic result (**d**). Given the extent of disease in the SVG, the venous conduit was not procedurally occluded during the PCI.

In addition to concerns of distal embolisation, no-reflow phenomenon, and periprocedural MI in SVG PCI, the rates of restenosis within SVG stents are disproportionately higher than native vessel PCI despite the advances in drug-eluting stent technology. The DIVA trial comparing drug-eluting stents (DES) to bare metal stents (BMS) in the treatment of de novo SVG lesions showed rates of target vessel revascularisation of 12% and 11%, respectively, within the 12 month follow up period [71]. This is consistent with meta-analysis data published in 2018 comparing DES to BMS in SVG lesions, with target vessel revascularisation rates of 25% and 26%, respectively, over a follow-up range of 18 to 60 months [72]. Interestingly, there were no statistically significant differences in MACE rates between DES and BMS in SVG PCI within the pooled studies. When an SVG has progressed to a CTO, percutaneous intervention to the venous conduit is not recommended due to exceedingly high failure rates. A study of 34 patients with chronic SVG occlusions reported that procedural success with stent implantation was low at only 68% [73]. Of those who achieved a successful stenting result, the rate of TVR and in-stent restenosis at 18-month follow-up was high at 61 and 68%, respectively. Degenerated or chronically-occluded SVG conduits can be used by experienced operators to perform retrograde recanalisation of the native coronary, however, with good procedural success rates [74] (Figure 2). While the feasibility of drug-eluting balloons (DEB) for de-novo graft lesions has been described in several small case series, their results have been variable and as such, require further evaluation before being adopted in wider practice [75,76].

### 5.5. Percutaneous Coronary Intervention to the Previously Bypassed Native Coronary Artery

The alternative to SVG PCI in failed grafts is PCI to the native vessel, ‘replacing’ the grafts and restoring patency within the previously grafted coronary artery. This strategy has additional challenges to de novo coronary artery PCI; however, due to the high burden of complex atherosclerotic lesion morphology, extensive coronary calcification, and the high incidence of CTO. Observational data suggest as high as 89% prevalence of CTO lesions in diagnostic angiography of patients with grafted coronary arteries [77]. These subsequently confer a lower procedural success rate [33]. Data from the RECHARGE registry comparing native vessel CTO PCI in patients with prior CABG versus no prior CABG revealed a significantly lower success rate in post-CABG patients (71.9% vs. 88.7%, *p* < 0.001), with results independent of the presence of a previous graft to the target CTO vessel [33]. Further multicentre international registry data collected from 2012 to 2023 comparing 12,164 patient outcomes following CTO PCI with or without the presence of previous CABG demonstrated similar findings, with lower procedural success in the prior CABG group (80.8% vs. 86.8%, *p* < 0.001) in addition to higher in-hospital mortality (0.8% vs. 0.3%, *p* < 0.001), acute myocardial infarction (0.9% vs. 0.5%, *p* = 0.007), and perforation (7.0% vs. 4.2%, *p* < 0.001) [32].

When undertaking native vessel PCI, the presence of a patent SVG to the treated native coronary artery segment has raised concerns that this may compromise the durability of the PCI result due to competitive flow. This is supported by observational data demonstrating areas of turbulent, non-linear flow and greater shear stress is associated with vascular dysfunction, accelerated atherosclerotic plaque formation, and subsequent early restenosis and stent thrombosis [78,79,80,81]. The safety and feasibility of SVG ‘sacrifice’ using percutaneous vascular occlusive devices during the PCI procedure has been demonstrated in case series; however, further randomised data are needed to guide whether this should be considered routine in native vessel PCI post CABG [82].

Irrespective of the presence of a CTO or the adoption of SVG sacrifice techniques, large observational studies have reported worse short- and long-term outcomes with SVG PCI compared to native vessel PCI [83,84,85]. These studies demonstrate a higher in-hospital mortality and higher rates of postprocedural complications in SVG PCI compared with native artery PCI [83]. Additionally, patients in the SVG PCI groups were more likely to require intra-aortic balloon pump counter pulsation; have longer fluoroscopy time, larger amounts of contrast, and lower TIMI flow grade; and are more likely to require a blood transfusion. Based on this observational data, European guidelines support PCI of the bypassed native artery over the bypass graft with Level IIa (Class C) evidence [2]. Despite these known limitations, SVG PCI is still observed to be performed in up to 37% of patients with previous CABG and SVG failure [83].

## 6. Future Directions

To date, randomised controlled trials comparing SVG PCI to native vessel PCI in graft failure have not been performed. The PROCTOR study (percutaneous coronary intervention of native coronary artery versus bypass graft in patients with prior CABG), commenced in 2019 and estimated to complete in 2027, is the first large randomised controlled trial designed to investigate this question [86]. The study aims to recruit 584 participants with a 1:1 randomisation to native versus SVG PCI, with potential native CTOs managed per the hybrid algorithm by experienced operators. Distal protection devices for SVG PCI will be used at the discretion of the operator. The primary endpoint of this study is MACE at three years. These results are eagerly awaited to provide further guidance on optimal percutaneous management in SVG failure.

## 7. Conclusions

Given the high prevalence of SVG use in CABG worldwide, the challenges of SVG failure will continue to be an important management issue for both cardiac surgeons and cardiologists. While redo CABG is a feasible option in some circumstances, its high operative and in-hospital mortality risk has made percutaneous revascularisation strategies the far preferred management option in these patients. Increasing evidence demonstrating the rapid progression of not only the venous conduit but also the native bypassed coronary artery has increased our understanding of the complexity of subsequent interventions. Both SVG PCI and native vessel PCI are substantive options in SVG failure when revascularisation is required, with each providing unique challenges to the interventional cardiologist. While data on the optimum strategy are currently observational only, the results of the PROCTOR trial will provide additional guidance on the safest and most effective percutaneous management for SVG failure.

## Figures and Tables

**Table 1:** Saphenous venous graft failure rates post index CABG procedure.

	SVG Failure Rates Post Index CABG	References
Less than 3 years	11–41%	[8,9,13,14]
5 to 10 years	19–33%	[10,15,16,17]
Greater than 10 years	39–61%	[10,11,22,23,24]

**Table 2:** Repeat revascularisation rates post index CABG procedure.

	Repeat Revascularisation Rates Post Index CABG Procedure	References
1 year	2%	[36]
5 years	7%	[36]
10 years	13%	[36]
18 years	16%	[36]

## Data Availability

Not applicable.
